# Dysfunction of Gpl1–Gih35–Wdr83 Complex in *S. pombe* Affects the Splicing of DNA Damage Repair Factors Resulting in Increased Sensitivity to DNA Damage

**DOI:** 10.3390/ijms25084192

**Published:** 2024-04-10

**Authors:** Ingrid Cipakova, Matus Jurcik, Tomas Selicky, Laura Olivia Lalakova, Jana Jakubikova, Lubos Cipak

**Affiliations:** 1 Department of Genetics, Cancer Research Institute, Biomedical Research Center, Slovak Academy of Sciences, Dubravska cesta 9, 84505 Bratislava, Slovakia; matus.jurcik@savba.sk (M.J.); tomas.selicky@savba.sk (T.S.); lauraolivia.lalakova@savba.sk (L.O.L.); 2 Department of Tumor Immunology, Cancer Research Institute, Biomedical Research Center, Slovak Academy of Sciences, Dubravska cesta 9, 84505 Bratislava, Slovakia; jana.jakubikova@savba.sk

**Keywords:** Gpl1, Gih35, Wdr83, G-patch protein, pre-mRNA splicing, DNA damage, *S. pombe*

## Abstract

Pre-mRNA splicing plays a key role in the regulation of gene expression. Recent discoveries suggest that defects in pre-mRNA splicing, resulting from the dysfunction of certain splicing factors, can impact the expression of genes crucial for genome surveillance mechanisms, including those involved in cellular response to DNA damage. In this study, we analyzed how cells with a non-functional spliceosome-associated Gpl1–Gih35–Wdr83 complex respond to DNA damage. Additionally, we investigated the role of this complex in regulating the splicing of factors involved in DNA damage repair. Our findings reveal that the deletion of any component within the Gpl1–Gih35–Wdr83 complex leads to a significant accumulation of unspliced pre-mRNAs of DNA repair factors. Consequently, mutant cells lacking this complex exhibit increased sensitivity to DNA-damaging agents. These results highlight the importance of the Gpl1–Gih35–Wdr83 complex in regulating the expression of DNA repair factors, thereby protecting the stability of the genome following DNA damage.

## 1. Introduction

The process of pre-mRNA splicing is essential for regulating gene expression in eukaryotes. During the splicing, the pre-mRNAs are processed to ensure the removal of noncoding sequences (introns) and the joining of the coding sequences (exons) to produce the mature messenger RNAs (mRNAs) encoding functional proteins [1,2,3].

Several evidences suggest that mutations in spliceosome components or their dysregulation are associated with defects in pre-mRNA splicing. For example, mutation in serine-arginine rich splicing factor SRSF2 was found to change its binding affinity from G-rich motifs to C-rich motifs. This causes the retention of introns in the integrator complex subunit 3 (INTS3) transcript, generating its isoforms with premature termination codons [4,5,6]. Other splicing factors, that deregulation is known to alter pre-mRNA splicing, are heterogeneous nuclear ribonucleoproteins (hnRNPs). The upregulation of hnRNPs allows them to bind to their own transcripts, promoting skipping of exons and generating hnRNPs isoforms with premature termination codons [7,8].

Except for the functions of splicing factors in pre-mRNA splicing, some splicing factors also have functions unrelated to splicing. For example, a depletion of the well-recognized splicing factor SRSF1 has been found to have a hypermutagenic phenotype, causing the accumulation of R-loops, which are structures considered to be one of the major threats to genome stability [9,10,11]. It has been revealed that the interaction of SRSF1 with DNA repair factor FANCD2 plays a critical role in suppressing the formation of R-loops via mRNA export regulation [12]. Similarly, the spliceosome-associated protein Nrl1 in *S. pombe* has been found to be required for the suppression of homologous recombination-dependent R-loop formation [13]. Another splicing factor interacting with DNA repair proteins is the G-patch protein NTR1. Its interaction with XRCC4 was shown to abrogate the formation of an active enzyme complex between XRCC4 and LIG4, thus reducing the DNA damage repair by non-homologous end joining pathway (NHEJ) [14]. It was also shown that the interactions between the splicing factor SFPQ and RAD51 recombinase, TopBP1 and Matrin3 secure their recruitment to sites of DNA damage thus stimulating DNA damage repair by NHEJ and homologous recombination [15,16,17,18].

Among the numerous factors involved in pre-mRNA splicing, the RNA helicases have a specific position. In addition to their role as ATP-dependent motors that drive transitions between spliceosomal complexes, they also control fidelity of splicing through kinetic proofreading [19]. For example, Prp22 helicase competes with the exon ligation reaction by pulling on the 3′ exon in the spliceosomal C* complex [20]. Furthermore, Prp43 helicase, in complex with Ntr1 and Ntr2, can disassemble early spliceosomal complexes formed on suboptimal substrates [21]. As RNA helicases play central roles in most processes of pre-mRNA splicing, their activities are tightly regulated.

The specific cofactor proteins regulating RNA helicase are the G-patch proteins. These proteins are characterized by the presence of a specific glycine-rich 45–50 amino acids long G-patch domain [22,23,24]. It has been shown that G-patch proteins can directly interact with RNA helicases. For example, in *S. cerevisiae*, Cmg1, Ntr1, Pxr1 and Sqs1 bind to helicase Prp43, and Spp2 associates with Prp2 helicase [21,22,25,26,27,28]. In human cells, the G-patch proteins CMTR1, GPATCH2, NKRF, PINX1, RBM5, RBM17, TFIP11 and ZGPAT interact with DHX15 helicase and GPKOW interact with DHX16 helicase [29,30,31,32]. In *S. pombe*, the G-patch proteins Ntr1 and Gpl1 were found to interact with Prp43 and Gih35 helicases, respectively [33,34,35]. Concerning the G-patch proteins and their role in regulating pre-mRNA splicing, it has been shown that Spp2 is required for spliceosome activation prior to the first transesterification reaction [28,36]. On the other hand, the Ntr1 protein was found to potentiate the activity of Prp43 helicase within the spliceosomal NTR complex [21,37,38]. It has also been revealed that G-patch proteins modulate domain motility of Prp43 helicase by inducing an open conformation of its RecA domains, thus facilitating ADP release and enabling processive translocation and unwinding [39,40]. Furthermore, it has been found that dysfunction of G-patch protein GPKOW, which interacts with DHX16 helicase, results in impairment of pre-mRNA splicing [32,41]. Finally, the interacting partners of DHX15 helicase, the G-patch proteins SUGP1, RBM5, RBM17 or ZGPAT, have been found to participate in correct branch site recognition, regulation of alternative splicing or in the regulation of tri-snRNP maturation [24,42,43,44,45]. 

Despite these findings, our current understanding of the biological significance of G-patch proteins in the regulation of RNA helicases is still limited, and further studies are needed. Recently, we reported that the poorly characterized G-patch protein Gpl1 in the fission yeast *S. pombe* forms a ternary complex with RNA helicase Gih35 and WD repeat protein Wdr83. We showed that Gpl1 binds to both Gih35 and Wdr83, allowing the association of these two proteins with the spliceosome. Additionally, we found that dysfunction of the Gpl1–Gih35–Wdr83 complex affects the efficiency of pre-mRNA splicing [35]. Importantly, the study by Larson et al. proposed that a depletion of Gpl1 in *S. pombe* results in global splicing defects [46].

Here, we present the results of a study designed to better characterize the importance of the Gpl1–Gih35–Wdr83 complex in regulating pre-mRNA splicing and the maintenance of genome stability in *S. pombe*. We tested the sensitivity of deletion mutants of this complex to DNA damage and analyzed the efficiency of splicing of genes encoding DNA damage repair factors.

## 2. Results and Discussion

### 2.1. Dysfunction of the Gpl1–Gih35–Wdr83 Complex Does Not Affect the Response of Mutant Cells to Acute Hydroxyurea (HU) Treatment but Makes These Cells Highly Sensitive to Chronic HU Treatment and DNA Damage

We have previously reported that the G-patch protein Gpl1, the RNA helicase Gih35 and the WD repeat protein Wdr83 in the fission yeast *S. pombe* form a ternary Gpl1–Gih35–Wdr83 complex that binds to the spliceosome. Further analysis revealed that removing the components of this complex leads to inefficient splicing and increased intron retention in selected reference genes [35]. Previously, mutants with a deletion of *gpl1* have been shown to exhibit canonical splicing defects, with broad increases in pre-mRNA isoforms and decreases in mature mRNAs [46].

**Figure 1:**
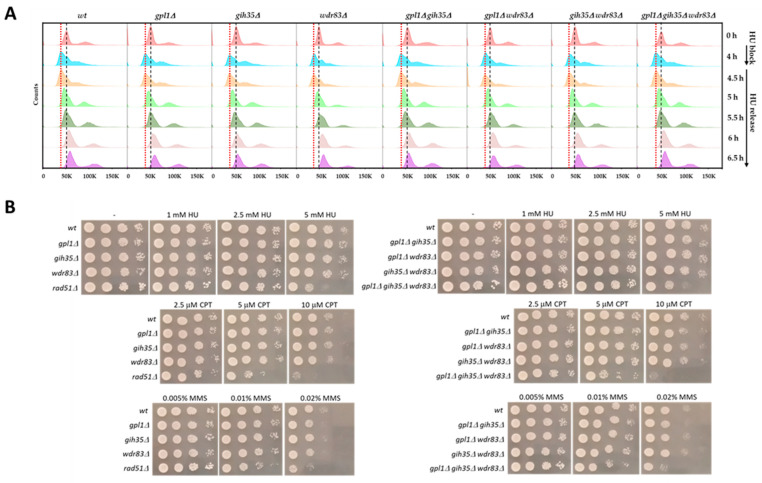
Analysis of the response of deletion mutants of the Gpl1–Gih35–Wdr83 complex to DNA damage. (**A**) Cell cycle analysis of the progression of cells through the cell cycle after acute hydroxyurea (HU) treatment (block of cells in G_1_/S phase using 12 mM HU), followed by releasing the cells from the block by growing cells in the fresh YE + 5S media (HU release). Red dot lines indicate the cells with a 1C DNA content and black dashed lines indicate the cells with a 2C DNA content. Exponentially growing asynchronous cells (0 h) represent the cells before the HU treatment. (**B**) The sensitivity of wild-type cells (*wt*) and deletion mutants of *gpl1*, *gih35* and *wdr83* to various DNA-damaging agents was examined by the standard spot test. The *rad51*∆ mutant was used as a positive control. Serially diluted cells were spotted onto standard YE + 5S plates and YE + 5S plates supplemented with indicated concentrations of HU, CPT or MMS, and incubated for 4 days at 32 °C. *Note:* The results shown are from one experiment. The data are representative of two independent experiments.

Since dysfunction of certain splicing factors can affect the expression and splicing of genes involved in the maintenance of genome stability [47,48,49,50], we decided to analyze how the deletion mutants of the Gpl1–Gih35–Wdr83 complex respond to DNA damage. First, we assessed the sensitivity of these mutants to acute hydroxyurea (HU) treatment. We found that in the presence of HU, the wild-type cells were arrested at the G_1_/S phase in 4 h (Figure 1A, 4 h point of HU block). Under similar conditions, single *gpl1*∆, *gih35*∆ and *wdr83*∆, double *gpl1*∆ *gih35*∆, *gpl1*∆ *wdr83*∆, and *gih35*∆ *wdr83*∆ and triple *gpl1*∆ *gih35*∆ *wdr83*∆ mutants, were also arrested in G_1_/S phase in 4 h. After releasing the cells from HU block, both the wild-type cells and deletion mutants behave similarly and completed the bulk of DNA synthesis in 1.5 to 2 h after release from HU block (Figure 1A, 5.5 h and 6 h points of HU release). These results suggest that mutants of the Gpl1–Gih35–Wdr83 are relatively insensitive to acute HU treatment, and after acute HU treatment, they arrest in the G_1_/S phase of the cell cycle similarly to wild-type cells. Furthermore, the HU block and release experiment suggest that the DNA replication checkpoint, which plays an important role in the response of cells to the HU-induced replication stress [51,52], is fully functional in the mutants of the Gpl1–Gih35–Wdr83 complex.

Despite the relative insensitivity of the deletion mutants of the Gpl1–Gih35–Wdr83 complex to acute HU treatment, we next investigated their sensitivity to chronic HU exposure and DNA damage caused by camptothecin (CPT), a drug which targets topoisomerase I, and DNA alkylating agent methyl methanesulfonate (MMS) [53,54]. As presented, the single *gpl1*∆, *gih35*∆ and *wdr83*∆ or double *gpl1*∆ *gih35*∆, *gpl1*∆ *wdr83*∆ and *gih35*∆ *wdr83*∆ mutants were insensitive to DNA damage induced by HU and CPT compared with wild-type cells. In the case of MMS, mild sensitivities of single *gpl1*∆ and *wdr83*∆, or double *gpl1*∆ *gih35*∆ mutants, were detected. Interestingly, the *gpl1*∆ *gih35*∆ *wdr83*∆ mutant, was shown to be highly sensitive to all tested DNA-damaging agents (Figure 1B). We found that the sensitivity of the triple deletion mutant of the Gpl1–Gih35–Wdr83 complex to DNA damaging agents was comparable to the *rad51*∆ mutant. It is known that *rad51* mutants are highly sensitive to DNA lesions induced by MMS, γ- or UV-irradiation. Rad51 is recombinase that forms nucleoprotein filaments on resected single-stranded DNA tails and catalyzes DNA strand exchange with undamaged sister chromatids, which serve as the templates for homologous recombination repair [55,56,57,58].

Although we did not detect any DNA repair factors to copurify within the Gpl1–Gih35–Wdr83 complex, we cannot exclude the possibility that dysfunction of the Gpl1–Gih35–Wdr83 complex affects DNA damage repair indirectly. Previously, we found that this complex copurifies with the Nrl1 protein and, conversely, that the Nrl1 protein copurifies within the Gpl1–Gih35–Wdr83 complex. We have shown that the spliceosome-associated Nrl1 protein plays an important role in suppressing the accumulation of genome-threatening R-loops. We also found that the deletion of *nrl1* increases the susceptibility of *S. pombe* cells to DNA damage, likely due to the sequestration of DNA repair factors near R-loop sites [13,34]. Despite these interesting findings, further studies are needed to demonstrate the existence of a functional link between the Gpl1–Gih35–Wdr83 complex and the Nrl1 protein.

Based on the findings that dysfunction of the Gpl1–Gih35–Wdr83 complex leads to global splicing defects and the accumulation of improperly spliced pre-mRNA [35,47], we hypothesize that dysfunction of the Gpl1–Gih35–Wdr83 complex might also affect splicing of DNA damage repair genes. This, in turn, could affect the ability of deletion mutants of the Gpl1–Gih35–Wdr83 complex to cope with DNA damage.

### 2.2. Dysfunction of the Gpl1–Gih35–Wdr83 Complex Affects the Splicing of DNA Damage Repair Genes

To analyze if the increased sensitivity of cells with a dysfunctional Gpl1–Gih35–Wdr83 complex to DNA-damaging agents is related somehow to the ability of these cells to process the pre-mRNA of genes participating in the maintenance of the genome stability, we evaluated the splicing efficiency of several genes, focusing in particular on *ssb1*, *ssb2*, *ssb3*, *swi5*, *rad16* and *rad57*, which encode DNA damage repair factors. It is known that DNA replication factor A subunits Ssb1, Ssb2 and Ssb3, and DNA repair endonuclease XPF Rad16 participate in double-strand break repair via homologous recombination, nucleotide excision repair, base-excision repair or mismatch repair. Additionally, recombination mediator Swi5 and RecA family ATPase Rad57 participate in the regulation of DNA recombination, which is important for DNA damage repair [59,60,61,62,63,64,65].

**Figure 2:**
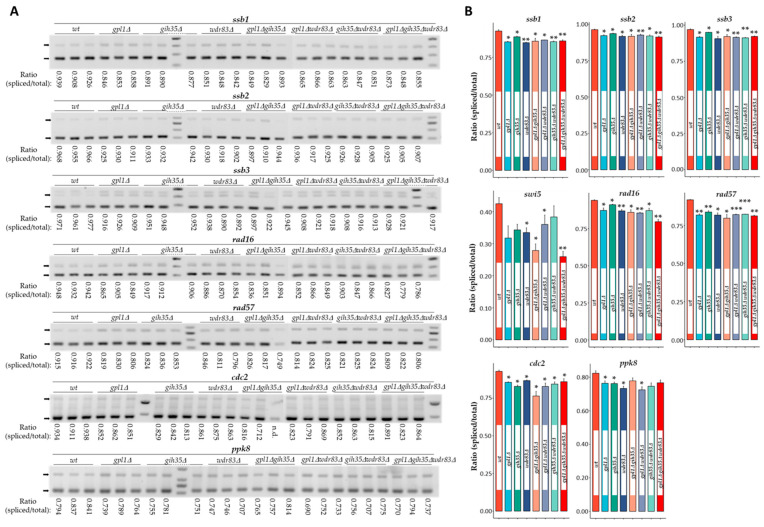
Analysis of splicing efficiency in deletion mutants of the Gpl1–Gih35–Wdr83 complex. (**A**) RT-PCR experiment showing spliced mRNAs (smaller PCR amplicon marked with arrow) and unspliced pre-mRNAs (larger PCR amplicon marked with arrow) of *ssb1*, *ssb2*, *ssb3*, *swi5*, *rad16, rad57*, *cdc2* and *ppk8* genes. (**B**) Analysis of the splicing efficiency of analyzed genes. The changes in the amount of unspliced pre-mRNA and spliced mRNA for analyzed genes were quantified using ImageJ software, version number 1.53q, and are expressed as the ratio of spliced mRNA (intensity of smaller PCR amplicon) versus total mRNA (intensities of spliced mRNA and unspliced pre-mRNA, smaller and larger PCR amplicons). The primers to amplify the splicing products of analyzed genes are listed in Supplemental Table S1. The data represent mean values ± S.D. from three independent biological replicates. Statistical significance between wild-type cells and mutants was determined using the two-tailed Student’s *t*-test (*p*-values: *—*p* ≤ 0.05, **—*p* ≤ 0.01, ***—*p* ≤ 0.001).

By analyzing deletion mutants of the Gpl1–Gih35–Wdr83 complex, we found that these mutants exhibit splicing defects in genes encoding DNA damage repair factors. Evaluating the ratio of spliced mRNA to total mRNA, we observed a significant decrease in the amount of properly spliced mRNA for all tested DNA damage repair genes. We detected a decreased amount in spliced mRNA ranging from 4.2 to 8.4% for *ssb1*, 2.9 to 5.3% for *ssb2*, 2.0 to 6.5% for *ssb3*, 9.7 to 38.7% for *swi5*, 3.1 to 8.4% for *rad16* and 8.8 to 13.2% for *rad57* (Figure 2A,B).

Similarly, analysis of the splicing of *ppk8* and *cdc2*, genes encoding the serine/threonine protein kinase Ppk38 and the cyclin-dependent protein kinase Cdc2 [66,67], which were used as controls of pre-mRNA splicing in the deletion mutants of the Gpl1–Gih35–Wdr83 complex, revealed an important role for the Gpl1–Gih35–Wdr83 complex in the regulation of pre-mRNA splicing. In this case, we detected an accumulation of improperly spliced pre-mRNA isoforms of *ppk8* and *cdc2*, and a decreased ratio in spliced mRNA ranging from 7.2 to 18.2% for *cdc2* and 5.5 to 12.0% for *ppk8* (Figure 2A,B). A similar, albeit more pronounced, decreased ratio in properly spliced pre-mRNA was detected in the *saf5*∆ mutant, which was used as a positive control for splicing defects when analyzing the splicing of *ssb1* (31.3%), *ssb2* (20.5%), *ssb3* (17.9%), *swi5* (47.4%), *rad16* (51.6%), *rad57* (13.8%), *cdc2* (9.9%) and *ppk8* (4.7%) (Figure S1). Saf5 factor of *S. pombe* is known to be critical for efficient snRNP production and pre-mRNA splicing [68].

Overall, analysis of splicing efficiency in deletion mutants of the Gpl1–Gih35–Wdr83 complex revealed significant splicing defects. This is evidenced by the accumulation of pre-mRNA isoforms for all analyzed genes, including those encoding DNA damage repair factors.

Our findings suggest that *S. pombe* cells with a non-functional Gpl1–Gih35–Wdr83 complex accumulate improperly spliced pre-mRNA isoforms. It is known that an increase in the rate of intron retention can have significant downstream effects on gene expression, protein functions and cellular processes, including the alteration of mRNA stability and the generation of non-functional or aberrant proteins that interfere with the function of normal proteins. Previously, it has been shown that the MTREC complex, which specifically binds to cryptic unstable transcripts, meiotic mRNAs or improperly spliced pre-mRNA transcripts, interacts with the nuclear exosome and cooperates with various RNA-binding proteins and RNA-processing complexes to target improperly spliced RNAs for degradation [69]. Interestingly, our previous studies on the interactomes of Gpl1, Gih35 and Wdr83 proteins identified Mtl1 and Ctr1 proteins of the MTREC complex, Nrl1 protein, as well as the intron-specific pre-mRNA splicing-ubiquitin fusion protein Sde2, to copurify as part of their interactomes [34,35]. This might suggest that the functions of the Gpl1–Gih35–Wdr83 complex are interconnected with the regulation of these proteins, either by regulating the Sde2 protein, which participates in the excision of introns [62,63], by participating in regulation of the Nrl1 protein, which suppresses accumulation of genome-threatening R-loop structures [13] or by modulating the function of proteins of the MTREC complex, which ensure the timely degradation of improperly spliced RNAs by the nonsense-mediated RNA decay (NMD) pathway [70,71]. To further support these hypotheses, additional studies investigating the interconnections between the Gpl1–Gih35–Wdr83 complex, other splicing factors and the proteins comprising the MTREC complex are warranted.

## 3. Materials and Methods

### 3.1. Yeast Strains and Primers

The *S. pombe* strains were cultured at 32 °C in a complete yeast extract medium (YE + 5S; 5.0 g/L yeast extract, 3.0% glucose, 0.1 g/L L-leucine, 0.1 g/L L-lysine hydrochloride, 0.1 g/L L-histidine, 0.1 g/L uracil and 0.15 g/L adenine sulfate). *S. pombe* strains carrying gene deletions were constructed as described previously [35,72]. Strains and primers used in this study are listed in Supplemental Table S1.

### 3.2. Testing Response of Cells to Acute Hydroxyurea Treatment

To test the response of cells to acute HU treatment, the overnight cultures of wild-type cells and deletion mutants were diluted into fresh YE + 5S media to OD_600_ = 0.2 and grown at 32 °C while shaking. After reaching OD_600_ = 0.55, HU was added to the liquid cell cultures at a final concentration of 12 mM. The cells were then incubated for 4 h to arrest them at the G_1_/S phase. Then, cultures were centrifuged (32 °C, 3000× *g*, 3 min), supernatants were discarded and cells were resuspended in fresh liquid YE + 5S media. At indicated time points, 500 μL of cell cultures were collected by centrifugation (3000× *g*, 1 min) and fixed in 1 mL of 70% ethanol (4 °C).

### 3.3. Flow Cytometry Analysis

The fixed cells (0.1 mL) were added to 3 mL of 50 mM sodium citrate buffer, incubated for 10 min and collected by centrifugation (3000× *g*, 3 min). Then, the cells were resuspended in 0.5 mL of 50 mM sodium citrate buffer supplemented with 0.1 mg/mL RNase and incubated at 37 °C for 2 h. Next, 0.5 mL of 50 mM sodium citrate buffer containing 2 μM SYTOX Green dye was added (final concentration of 1 μM) to stain the DNA. Flow cytometry measurements were performed using a FACS Canto II flow cytometer (Becton Dickinson, Franklin Lakes, NJ, USA). The SYTOX Green dye was excited with a 488 nm laser, and fluorescence emission was measured using a bandpass filter set of 530 nm. Forward/side light scatter characteristics were used to eliminate cell debris from the analysis. For each analysis, 10,000 cells were acquired. The flow cytometry results were analyzed using FlowJo^TM^ v10.8.1 software (BD Life Sciences, Franklin Lakes, NJ, USA). 

### 3.4. Drug Sensitivity

DNA damage drug sensitivity was determined by the standard spot test, which allows a chronic drug exposure of cells. Briefly, YE + 5S plates containing 1, 2.5, 5 mM hydroxyurea (HU), 2.5 μM, 5 μM, 10 μM camptothecin (CPT) and 0.005%, 0.01% and 0.02% methyl methanesulfonate (MMS) were freshly prepared 3 days before the experiment. Cells were grown on YE + 5S plates overnight, diluted in fresh YE + 5S media to OD_600_ = 0.2, and grown for 6 h at 32 °C. Then, cells were resuspended in sterile water, and their concentration was determined using a Burker chamber. After diluting the cells in sterile water in 10-fold steps, 6 μL of cell suspension was spotted onto standard YE + 5S plates and YE + 5S plates supplemented with HU, CPT and MMS. The plates were incubated for 4 days at 32 °C and then photographed.

### 3.5. Isolation of RNA and Preparation of cDNA

The cells were inoculated into 20 mL of overnight culture in YE + 5S media. The next morning, the overnight cultures were diluted into 50 mL of fresh YE + 5S media (OD_600_ = 0.2) and cultivated on a shaker (32 °C) until reaching the exponential phase (OD_600_ = 0.5–0.6). Then, cell cultures (26 mL) were harvested by centrifugation (3000× *g*, 4 °C), the collected cells were washed once with ice-cold DEPC water, and cell pellets were stored at −80 °C. Next, cell pellets were resuspended in 1×TE and broken by vortexing with glass beads. Total RNA was isolated using Thermo Fisher Scientific kits (GeneJET RNA Purification Kit; RapidOut DNA Removal Kit) (Thermo Fisher Scientific, Waltham, MA, USA). cDNA was prepared from 1 μg total RNA using the Lunascript RT SuperMix Kit (NEB, Ipswich, MA, USA) according to the manufacturer‘s instructions.

### 3.6. Analysis of Splicing Defects

To analyze the splicing defects, semiquantitative RT-PCR was used. The conditions for each pair of primers and corresponding gene were optimized to bind (T_m_) and amplify (number of cycles) the spliced mRNA and unspliced pre-mRNA for *cdc2*, *ppk8*, *rad16*, *rad57 ssb1*, *ssb2*, *ssb3* and *swi5* (Supplemental Table S1). Genomic DNA (gDNA) was used as a control to position the PCR products of unspliced pre-mRNA. Actin (*act1*) was used as an internal control to monitor the efficiency of the PCR amplification. The amount of spliced mRNA and unspliced pre-mRNA was quantified as intensities of corresponding PCR amplicons using ImageJ software (version number 1.53q). Briefly, the images of PCR amplicons were converted to a grey scale format. Then, the regions of PCR amplicons corresponding to unspliced pre-mRNAs and spliced mRNAs were selected using a rectangular selection tool and plotted. The intensities of the PCR amplicons were quantified by measuring the total area of their respective peaks. The total mRNA of a specific gene was calculated by summing the intensities of its unspliced pre-mRNA and spliced mRNA. Splicing efficiency was determined as the ratio of intensity of spliced mRNA and intensity of total mRNA (spliced mRNA and unspliced pre-mRNA).

### 3.7. Statistical Analysis

The statistical analysis was performed on three independent biological replicates. Results are presented as the mean values ± standard deviations. Statistical significance between wild-type cells and mutants was determined using a two-tailed Student’s *t*-test. Significance was defined as *p* < 0.05.

## 4. Conclusions

Despite the evidence that the Gpl1–Gih35–Wdr83 complex plays an important role in the regulation of pre-mRNA splicing in *S. pombe*, including the splicing of genes encoding DNA damage repair factors, our understanding of how this ternary complex is implicated in the regulation of pre-mRNA splicing is still limited. Further structural and mechanistic studies to determine how this complex participates in the dynamic rearrangements of the spliceosome and possibly regulates other aspects of pre-mRNA splicing, or how it affects the response of cell to DNA damage, are highly warranted.

## Data Availability

The data are contained within the article.

## Supplementary Materials

The following supporting information can be downloaded at https://www.mdpi.com/article/10.3390/ijms25084192/s1.
