# Supplementary material for: Dysfunction of Gpl1–Gih35–Wdr83 Complex in S. pombe Affects the Splicing of DNA Damage Repair Factors Resulting in Increased Sensitivity to DNA Damage
Source: Int J Mol Sci. 2024 Apr 10;25(8):4192. doi: 10.3390/ijms25084192 (PMC11049892; doi:10.3390/ijms25084192)
Supplement: Supplementary file 1 [file ijms-25-04192-s001.zip › Figure S1.pptx]

## Slide 1
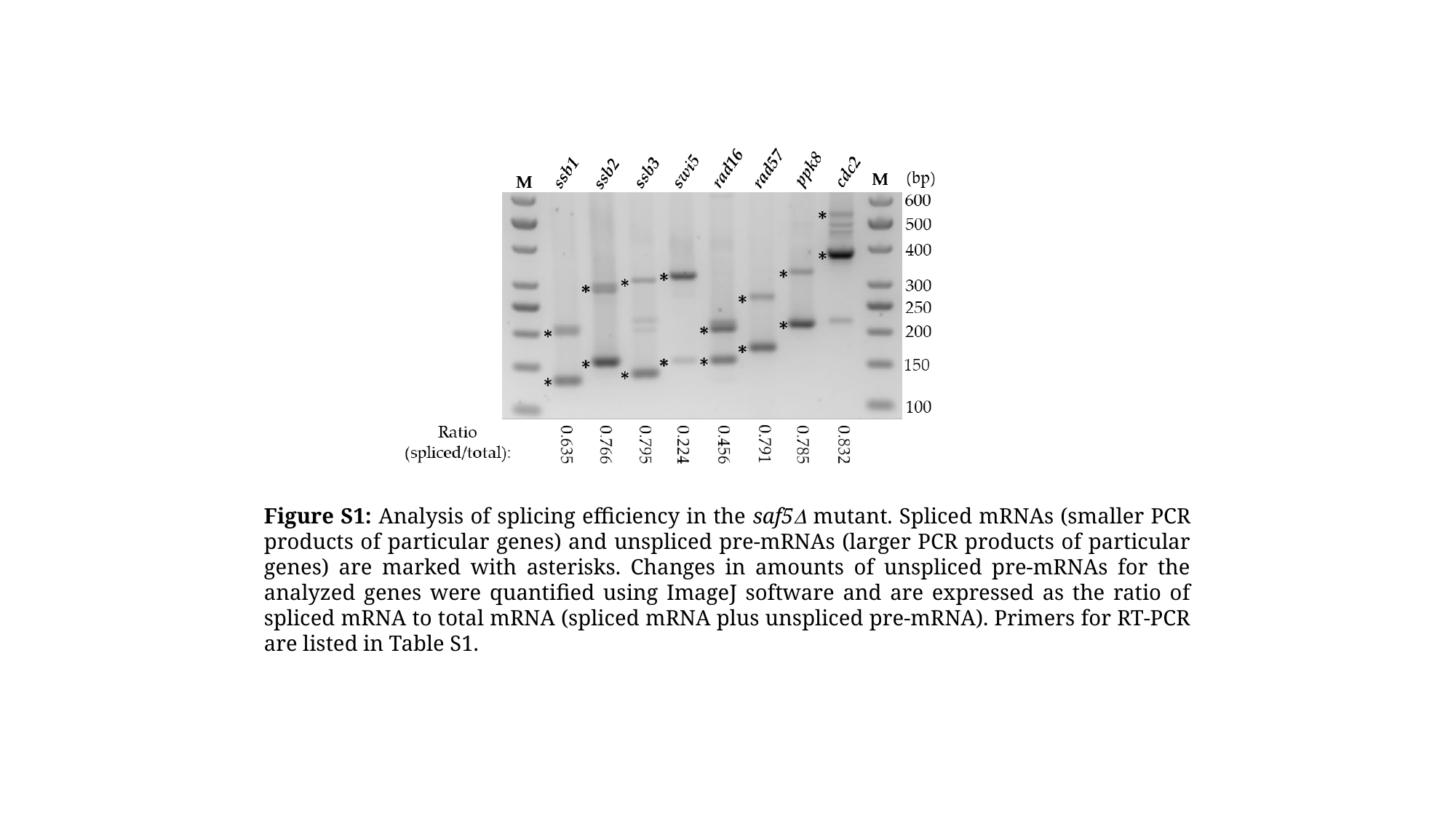

Figure S1: Analysis of splicing efficiency in the saf5D mutant. Spliced mRNAs (smaller PCR products of particular genes) and unspliced pre-mRNAs (larger PCR products of particular genes) are marked with asterisks. Changes in amounts of unspliced pre-mRNAs for the analyzed genes were quantified using ImageJ software and are expressed as the ratio of spliced mRNA to total mRNA (spliced mRNA plus unspliced pre-mRNA). Primers for RT-PCR are listed in Table S1.
